# Supplementary figures and images for: Evaluating Spatial Interaction Models for Regional Mobility in Sub-Saharan Africa
Source: PLoS Comput Biol. 2015 Jul 9;11(7):e1004267. doi: 10.1371/journal.pcbi.1004267 (PMC4497594; doi:10.1371/journal.pcbi.1004267)

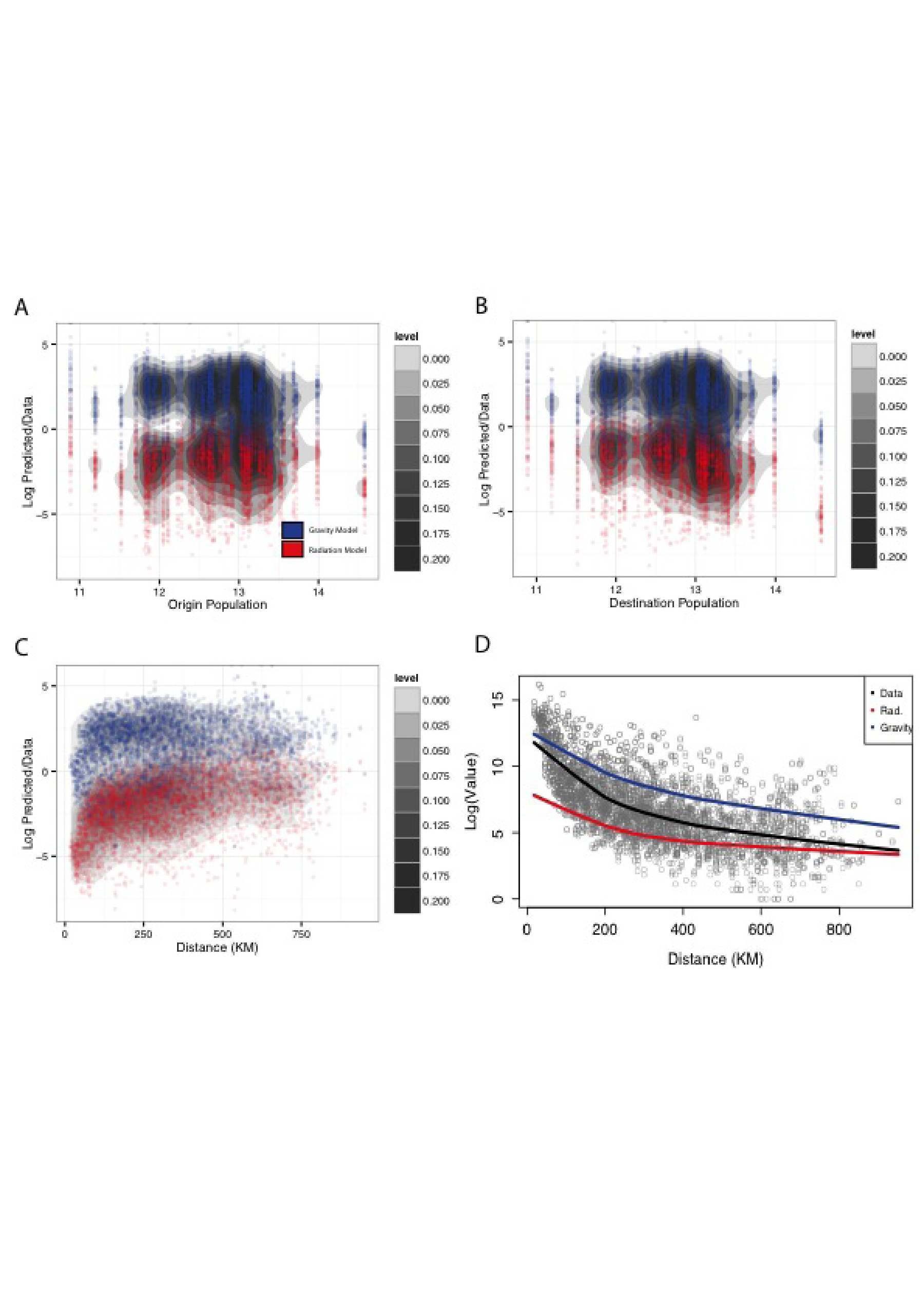

Supplement: S1 Fig — The relationship between the ratio of the predicted versus actual data is shown compared to the population A) of the origin, B) of the destination, and C) the distance between the origin and destination for all trips between districts. The gravity model consistently overpredicted travel, whereas the radiation model consistently underpredicted travel. For both the origin and destination population, there was no clear bias in the ability of each model to predict the volume of travel, although both models predicted more accurate estimates as the distance increased. D) The relationship between distance the amount of travel (log) with the trend line from predictions from the data (black), the gravity model (blue), and radiation model (red). (TIF) [file pcbi.1004267.s001.tif]

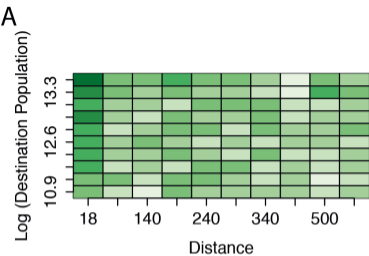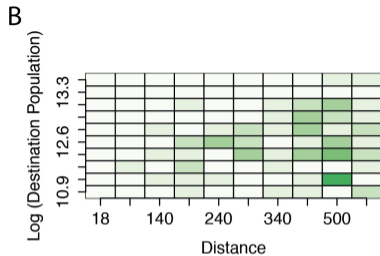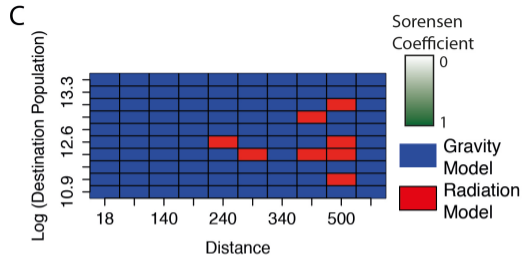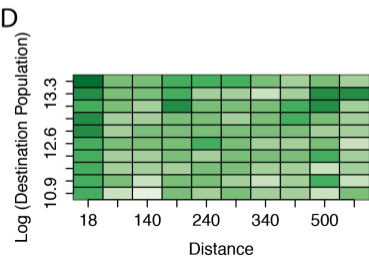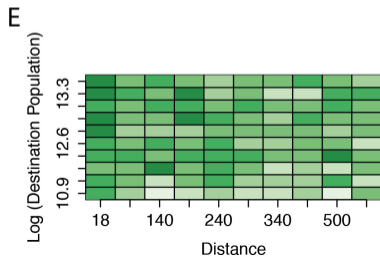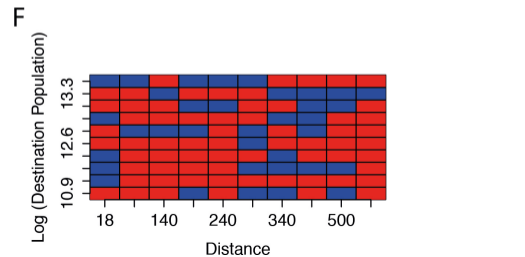

Supplement: S2 Fig — Using Euclidean distance, we compared the estimated versus empirical amount of travel between areas of varying population size and distance from the radiation and gravity models. We calculated a Sorensen-Dice coefficient to measure the difference between predicted and total volumes of travel. The coefficient values from a A) gravity model and B) radiation model are shown highlighting the better model performance of the gravity model. Both models performed well at predicting travel to nearby highly populated districts. In general, C) the gravity model outperformed the radiation model. We next compared the ability of both models to predict the relative amount of travel D) gravity model, F) radiation model. Both models performed better at predicting relative travel than the total volume of travel with G) the radiation model often outperforming the gravity model. (TIF) [file pcbi.1004267.s002.tif]

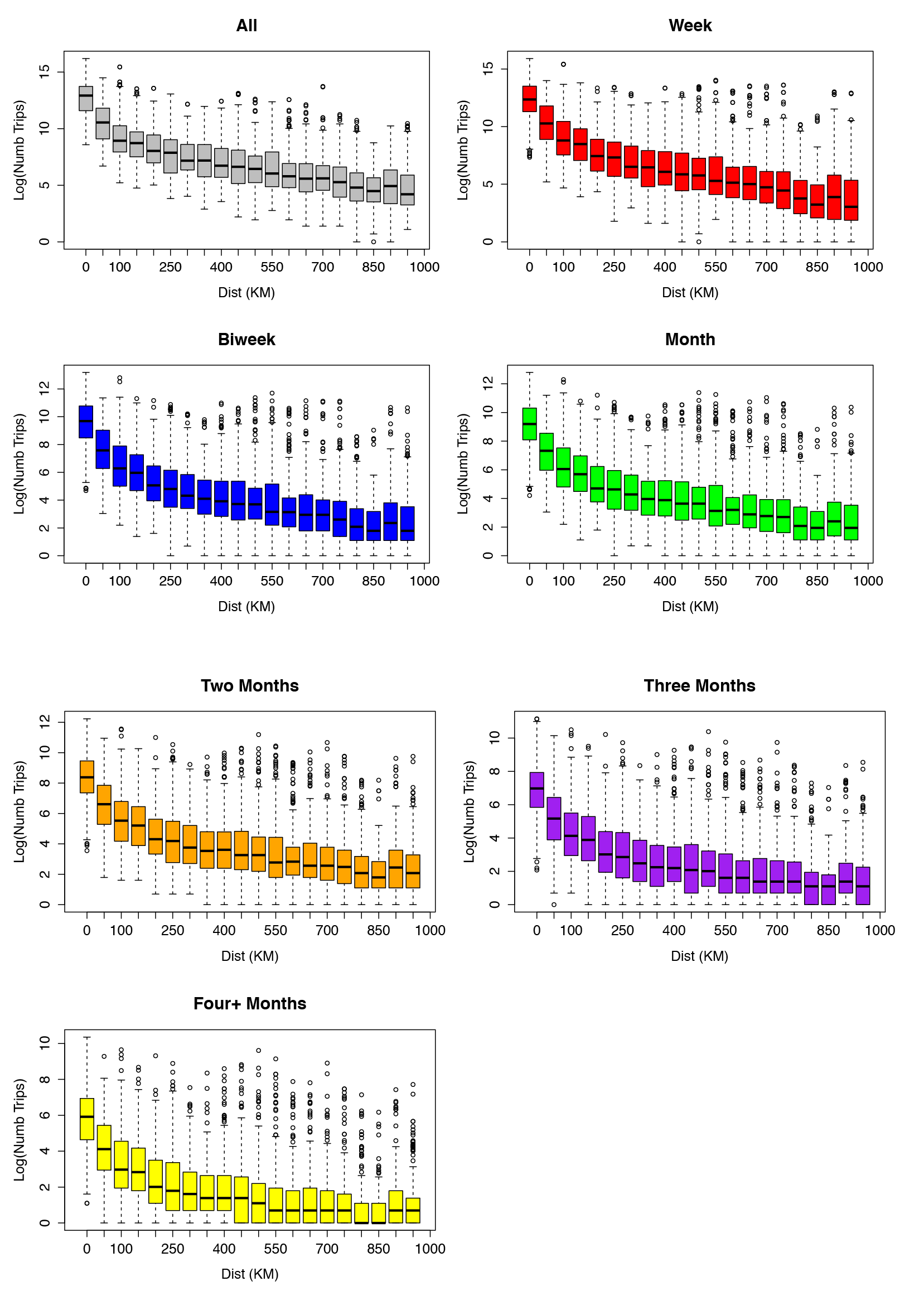

Supplement: S3 Fig — For all durations of travel, we compared the number of trips versus the Euclidean distance between the origin and destination. For all trip durations, the frequency of trips decays with geographic distance. As the duration of travel increases, the frequency of journeys decreases. (TIF) [file pcbi.1004267.s003.tif]

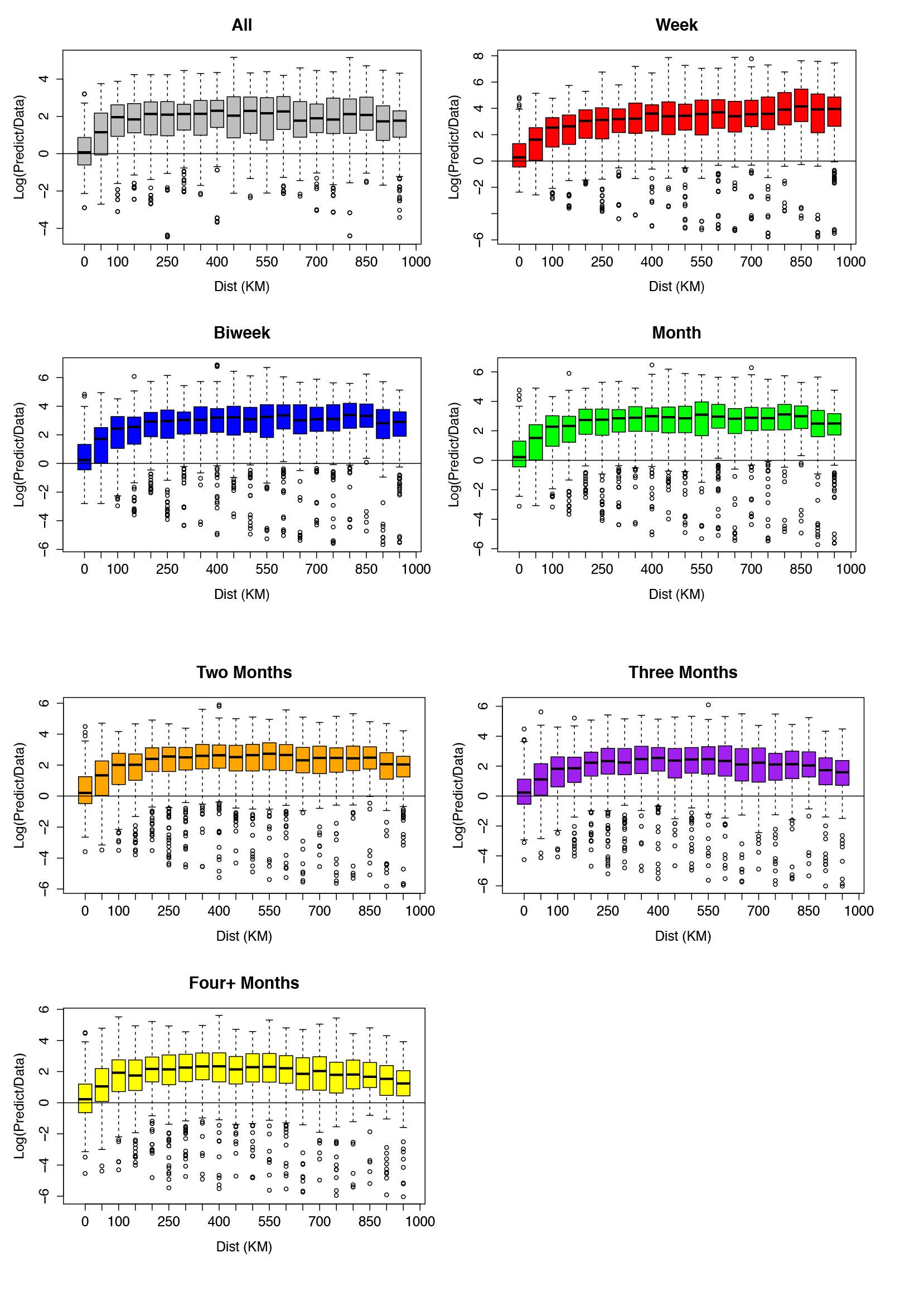

Supplement: S4 Fig — For each group of travel based on trip duration, we compared the gravity model predicted values (using Euclidean distance) versus the Euclidean distance between districts (in kilometers). In general, each gravity model over predicted travel and produced the most accurate estimates (log(predict/data) near 0) for travel over short geographic distances. (TIF) [file pcbi.1004267.s004.tif]

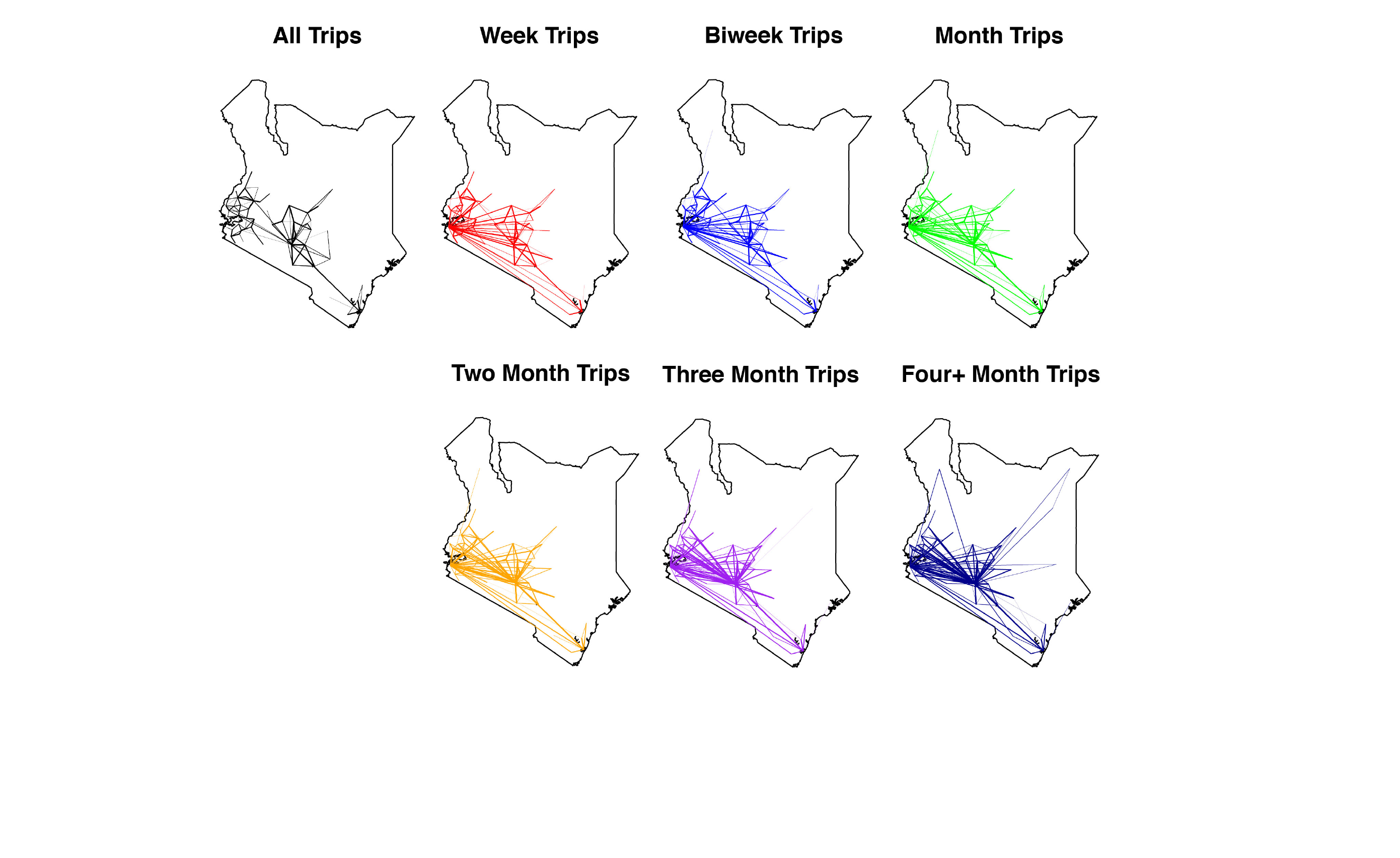

Supplement: S5 Fig — For each duration of travel, the top five percent of routes are shown. For trips lasting shorter durations, the most traveled routes are often to nearby districts. However, as the trip duration increases the most travel routes often include a major city such as Nairobi or Mombasa. (TIF) [file pcbi.1004267.s005.tif]

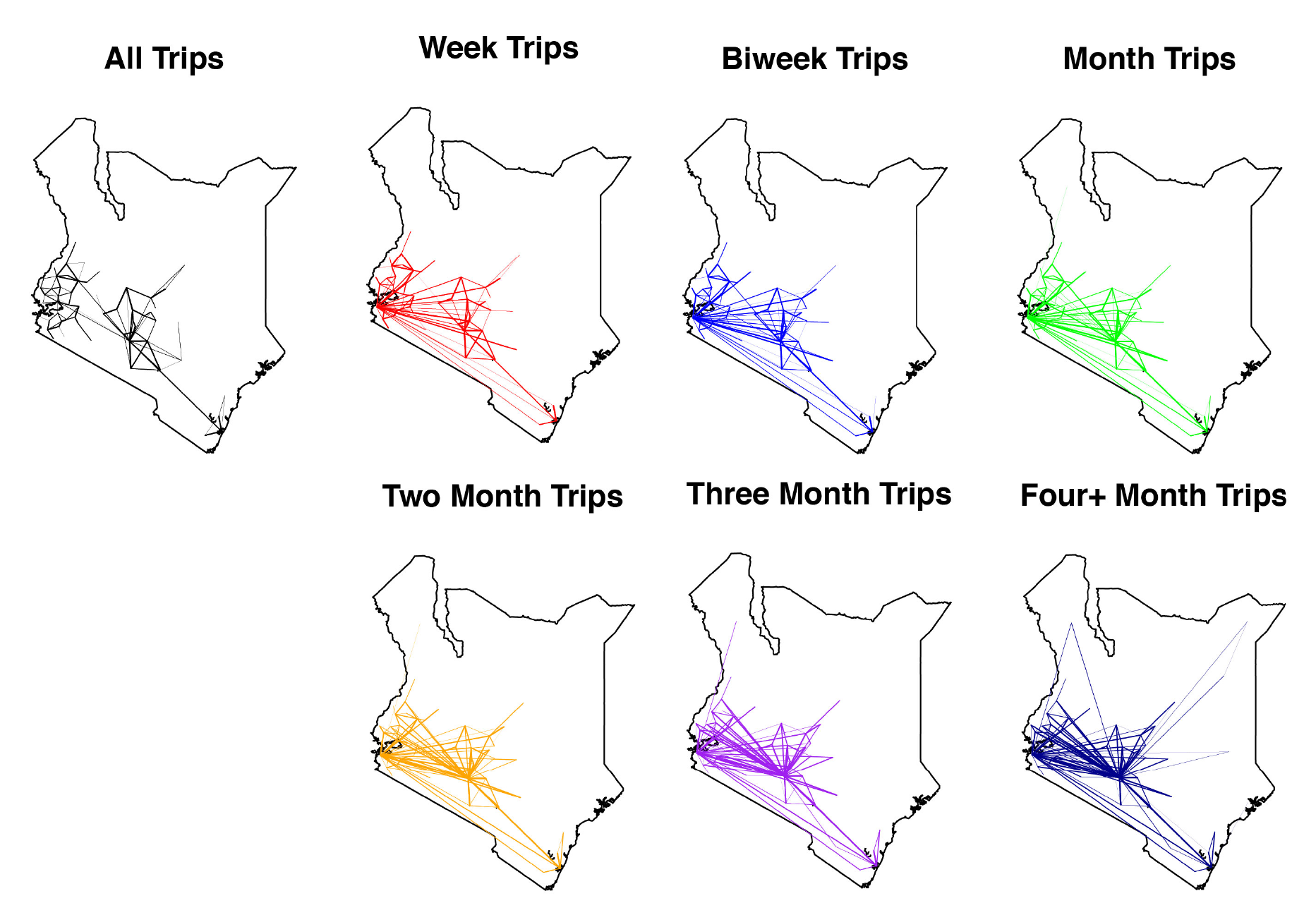

Supplement: S6 Fig — Similar to S5 Fig, we have plotted the top 200 routes of travel for various trip durations. As the trip duration increased, the most traveled routes often include a major city such as Nairobi or Mombasa. (TIF) [file pcbi.1004267.s006.tif]
