# Supplementary material for: Evaluating Spatial Interaction Models for Regional Mobility in Sub-Saharan Africa
Source: PLoS Comput Biol. 2015 Jul 9;11(7):e1004267. doi: 10.1371/journal.pcbi.1004267 (PMC4497594; doi:10.1371/journal.pcbi.1004267)
Supplement: S1 Table — Papers that included epidemiological disease data are labeled ‘D’ whereas those that completely simulated disease dynamics are labeled ‘S’. (DOCX) [file pcbi.1004267.s007.docx]

| **Table S1 A summary of the papers analyzed by disease.** Papers that included epidemiological disease data are labeled ‘D’ whereas those that completely simulated disease dynamics are labeled ‘S’. | | |
| --- | --- | --- |
| **Disease** | **Number of Papers** | **Reference** |
| Cholera | D: 2  S: | D: Gatto 2012; Mari 2011  S: |
| Malaria | D: 1  S: | D: Tatem 2006;  S: |
| Dengue | D: 1  S: 1 | D: Stoddard 2012  S: Vazquez-Prokopec 2013 |
| Measles | D: 2  S: | D: Ferrari 2008; Xia 2004  S: |
| Pertussis + Measles | D:  S: 1 | D:  S: Watts 2005 |
| Rubella | D: 1  S: | D: Metcalf 2013  S: |
| Influenza | D: 3  S: 8 | D: Gog 2013; Balcan 2009; Wang 2011  S: Truscott 2012; Tizzoni 2014; Balcan 2009; Dalziel 2013; Poletto 2012; Meloni 2011; Merler 2009; Mills 2014 |
| Foot and Mouth | D:  S: 1 | D:  S: Keeling 2010 |
