# Supplementary material for: Evaluating Spatial Interaction Models for Regional Mobility in Sub-Saharan Africa
Source: PLoS Comput Biol. 2015 Jul 9;11(7):e1004267. doi: 10.1371/journal.pcbi.1004267 (PMC4497594; doi:10.1371/journal.pcbi.1004267)
Supplement: S2 Table — (DOCX) [file pcbi.1004267.s008.docx]

| **Table S2 The gravity model parameters fit for subsets of the data.** | | | | |
| --- | --- | --- | --- | --- |
|  | Intercept ($k$) | Pop From ($\alpha$) | Pop To ($\beta$) | Dist ($\gamma$) |
| Full Data Set | -20.61 | 1.22 | 1.22 | -2.05 |
| To/From Nairobi | -12.66 | 0.95 | 0.95 | -1.65 |
| No To/From Nairobi | -20.61 | 1.22 | 1.22 | -2.05 |
| To/From Cities | -20.40 | 1.23 | 1.23 | -1.59 |
| No To/From Cities | -10.88 | 0.83 | 0.83 | -2.28 |
| Btwn Very Rural Areas | 2.37 | 0.38 | 0.37 | -3.70 |
| Btwn Med Rural Areas | -7.90 | 0.76 | 0.76 | -2.66 |
| From Very Rural Areas | -7.59 | -0.14 | 1.62 | -4.13 |
| From Med Rural Areas | -15.11 | 0.84 | 1.20 | -2.62 |
