# Supplementary material for: Evaluating Spatial Interaction Models for Regional Mobility in Sub-Saharan Africa
Source: PLoS Comput Biol. 2015 Jul 9;11(7):e1004267. doi: 10.1371/journal.pcbi.1004267 (PMC4497594; doi:10.1371/journal.pcbi.1004267)
Supplement: S3 Table — A: For the full data set, data including travel to/from: Nairobi, cities, and not including travel to/from: Nairobi, cities, the reduction in deviance (%) from fitting a gravity model is shown. For each distance measure, both population weighted centroids and non-population weighted centroids were calculated. B: For data including travel between or to/from very and moderately rural areas, the reduction in deviance (%) from fitting a gravity model is shown. For each distance measure, both population weighted centroids and non-population weighted centroids were calculated. (DOCX) [file pcbi.1004267.s009.docx]

| **Table S3 The reduction in deviance from a gravity model for subsets of the data using various distance measures.** Table S3A: For the full data set, data including travel to/from: Nairobi, cities, and not including travel to/from: Nairobi, cities, the reduction in deviance (%) from fitting a gravity model is shown. For each distance measure, both population weighted centroids and non-population weighted centroids were calculated. Table S3B: For data including travel between or to/from very and moderately rural areas, the reduction in deviance (%) from fitting a gravity model is shown. For each distance measure, both population weighted centroids and non-population weighted centroids were calculated.  **Table S3A** | | | | | |
| --- | --- | --- | --- | --- | --- |
|  | **Full Data Set** | **To/From Nairobi** | **No To/From Nairobi** | **To/From Cities** | **No To/From Cities** |
| Euclidean Distance Between Centroids | 80.06 | 87.27 | 80.06 | 87.11 | 76.58 |
| Travel Time Between Centroids | 43.50 | 64.27 | 43.50 | 55.01 | 28.74 |
| Road Distance Between Centroids | 78.70 | 85.99 | 78.70 | 86.09 | 74.73 |
| Euclidean Distance Between Pop Weight Centroids | 81.51 | 87.68 | 81.51 | 88.26 | 79.37 |
| Travel Time Between Pop Weight Centroids | 48.55 | 54.55 | 48.55 | 61.02 | 33.69 |
| Road Distance Between Pop Weight Centroids | 80.46 | 85.35 | 80.46 | 86.47 | 78.88 |

| **Table S3 The reduction in deviance from a gravity model for subsets of the data using various distance measures.** Table S3A: For the full data set, data including travel to/from: Nairobi, cities, and not including travel to/from: Nairobi, cities, the reduction in deviance (%) from fitting a gravity model is shown. For each distance measure, both population weighted centroids and non-population weighted centroids were calculated. Table S3B: For data including travel between or to/from very and moderately rural areas, the reduction in deviance (%) from fitting a gravity model is shown. For each distance measure, both population weighted centroids and non-population weighted centroids were calculated.  **Table S3B** | | | | |
| --- | --- | --- | --- | --- |
|  | **Btwn Very Rural Areas** | **Btwn Mod Rural Areas** | **From Very Rural Areas** | **From Mod Rural Areas** |
| Euclidean Distance Between Centroids | 63.25 | 79.78 | 83.20 | 77.15 |
| Travel Time Between Centroids | 46.29 | 37.65 | 38.20 | 36.64 |
| Road Distance Between Centroids | 72.23 | 78.78 | 81.17 | 76.08 |
| Euclidean Distance Between Pop Weight Centroids | 80.95 | 85.17 | 81.24 | 80.35 |
| Travel Time Between Pop Weight Centroids | 54.97 | 43.82 | 51.43 | 44.52 |
| Road Distance Between Pop Weight Centroids | 84.53 | 84.05 | 83.25 | 80.66 |
