# Supplementary material for: Evaluating Spatial Interaction Models for Regional Mobility in Sub-Saharan Africa
Source: PLoS Comput Biol. 2015 Jul 9;11(7):e1004267. doi: 10.1371/journal.pcbi.1004267 (PMC4497594; doi:10.1371/journal.pcbi.1004267)
Supplement: S4 Table — A: For data including travel between or to/from very and moderately rural areas, the Sorsensen-Dice coefficient from fitting a gravity model is shown. For each distance measure, both population weighted centroids and non-population weighted centroids were calculated. B: For data including travel between or to/from very and moderately rural areas, the Sorsensen-Dice coefficient from fitting a gravity model is shown. For each distance measure, both population weighted centroids and non-population weighted centroids were calculated. (DOCX) [file pcbi.1004267.s010.docx]

| **Table S4 The Sorsensen-Dice coefficient for subsets of the data using various distance measures.** Table S4A: For data including travel between or to/from very and moderately rural areas, the Sorsensen-Dice coefficient from fitting a gravity model is shown. For each distance measure, both population weighted centroids and non-population weighted centroids were calculated. Table S4B: For data including travel between or to/from very and moderately rural areas, the Sorsensen-Dice coefficient from fitting a gravity model is shown. For each distance measure, both population weighted centroids and non-population weighted centroids were calculated.  **Table S4A** | | | | | |
| --- | --- | --- | --- | --- | --- |
|  | **Full Data Set** | **To/From Nairobi** | **No To/From Nairobi** | **To/From Cities** | **No To/From Cities** |
| Euclidean Distance Between Centroids | 0.64 | 0.83 | 0.64 | 0.77 | 0.60 |
| Travel Time Between Centroids | 0.37 | 0.67 | 0.37 | 0.51 | 0.30 |
| Road Distance Between Centroids | 0.62 | 0.81 | 0.62 | 0.76 | 0.58 |
| Euclidean Distance Between Pop Weight Centroids | 0.63 | 0.79 | 0.63 | 0.76 | 0.62 |
| Travel Time Between Pop Weight Centroids | 0.42 | 0.61 | 0.42 | 0.58 | 0.32 |
| Road Distance Between Pop Weight Centroids | 0.61 | 0.77 | 0.61 | 0.73 | 0.60 |

| **Table S4 The Sorsensen-Dice coefficient for subsets of the data using various distance measures.** Table S4A: For data including travel between or to/from very and moderately rural areas, the Sorsensen-Dice coefficient from fitting a gravity model is shown. For each distance measure, both population weighted centroids and non-population weighted centroids were calculated. Table S4B: For data including travel between or to/from very and moderately rural areas, the Sorsensen-Dice coefficient from fitting a gravity model is shown. For each distance measure, both population weighted centroids and non-population weighted centroids were calculated.  **Table S4B** | | | | |
| --- | --- | --- | --- | --- |
|  | **Btwn Very Rural Areas** | **Btwn Med Rural Areas** | **From Very Rural Areas** | **From Med Rural Areas** |
| Euclidean Distance Between Centroids | 0.46 | 0.60 | 0.60 | 0.57 |
| Travel Time Between Centroids | 0.40 | 0.34 | 0.27 | 0.32 |
| Road Distance Between Centroids | 0.53 | 0.62 | 0.59 | 0.58 |
| Euclidean Distance Between Pop Weight Centroids | 0.66 | 0.68 | 0.57 | 0.62 |
| Travel Time Between Pop Weight Centroids | 0.44 | 0.34 | 0.33 | 0.33 |
| Road Distance Between Pop Weight Centroids | 0.74 | 0.63 | 0.59 | 0.63 |
