# Supplementary material for: Evaluating Spatial Interaction Models for Regional Mobility in Sub-Saharan Africa
Source: PLoS Comput Biol. 2015 Jul 9;11(7):e1004267. doi: 10.1371/journal.pcbi.1004267 (PMC4497594; doi:10.1371/journal.pcbi.1004267)
Supplement: S5 Table — (DOCX) [file pcbi.1004267.s011.docx]

| **Table S5 The ability of each model The ability of each model to capture various situations % (N).** | | | |
| --- | --- | --- | --- |
| **Error Less Equal 1** | **Gravity** | **Radiation** | **Eliminate** |
| Low Pop, Near | 22.2 (377) | 13.7 (233) | 64.1 (1700) |
| Low Pop, Far | 17.7 (314) | 36.9 (653) | 45.4 (1772) |
| High Pop, Near | 29.2 (500) | 11.8 (203) | 59 (1714) |
| High Pop, Far | 21.6 (355) | 38 (624) | 40.3 (1640) |
| High Volume | 37.5 (850) | 5 (114) | 57.4 (1300) |
| Low Volume | 7 (159) | 44.8 (1014) | 48.2 (2264) |
| **Error Less Equal 3** | **Gravity** | **Radiation** | **Eliminate** |
| Low Pop, Near | 55.6 (945) | 43.6 (742) | 0.8 (13) |
| Low Pop, Far | 37 (656) | 62.1 (1100) | 0.9 (16) |
| High Pop, Near | 60.6 (1039) | 38.9 (667) | 0.5 (8) |
| High Pop, Far | 39.4 (646) | 60.3 (989) | 0.3 (5) |
| High Volume | 21.5 (486) | 78.1 (1768) | 0.4 (10) |
| Low Volume | 74.6 (1689) | 24.5 (554) | 0.9 (21) |
| **Error Less Equal 4** | **Gravity** | **Radiation** | **Eliminate** |
| Low Pop, Near | 55.9 (951) | 43.7 (743) | 0.4 (6) |
| Low Pop, Far | 37.4 (662) | 62.2 (1103) | 0.4 (7) |
| High Pop, Near | 60.9 (1043) | 39 (669) | 0.1 (2) |
| High Pop, Far | 28.3 (464) | 60.4 (991) | 0 (0) |
| High Volume | 21.5 (486) | 78.3 (1773) | 0.2 (5) |
| Low Volume | 75.2 (1702) | 24.5 (554) | 0.4 (8) |
