# Supplementary material for: Evaluating Spatial Interaction Models for Regional Mobility in Sub-Saharan Africa
Source: PLoS Comput Biol. 2015 Jul 9;11(7):e1004267. doi: 10.1371/journal.pcbi.1004267 (PMC4497594; doi:10.1371/journal.pcbi.1004267)
Supplement: S6 Table — We fit a number of logistic regression equations using distance, the origin population, or destination population as the explanatory variable. For each regression equation (see above equations), the coefficients, intercept, and model fit is shown (percentage reduction in deviance and adjusted R2 value). (DOCX) [file pcbi.1004267.s012.docx]

| **Table S6 The regression results predicting when to use a gravity model or radiation model.** We fit a number of logistic regression equations using distance, the origin population, or destination population as the explanatory variable. For each regression equation (see above equations), the coefficients, intercept, and model fit is shown (percentage reduction in deviance and adjusted R^2^ value). | | | | | |
| --- | --- | --- | --- | --- | --- |
| **Variable** | **b_0_** | **b_1_** | **Reduc Dev (%)** | **Adj R^2^** | **p-value** |
| Distance  (per 100 KM) (Dist) | 0.673  (0.645, 0.702) | -0.609  (-0.068, -0.053) | 4 | 0.05826 | <0.001 |
| Origin Pop  (Origin) | -0.388  (-0.497, -0.278) | 0.0063  (0.004, 0.008) | 0.6 | 0.008423 | <0.001 |
| Destination Pop  (Dest) | -0.813  (-0.94, -0.689) | 0.017  (0.014, 0.02) | 0.3 | 0.04246 | <0.001 |
