# Supplementary material for: Evaluating Spatial Interaction Models for Regional Mobility in Sub-Saharan Africa
Source: PLoS Comput Biol. 2015 Jul 9;11(7):e1004267. doi: 10.1371/journal.pcbi.1004267 (PMC4497594; doi:10.1371/journal.pcbi.1004267)
Supplement: S1 Text — (DOCX) [file pcbi.1004267.s014.docx]

**Evaluating spatial interaction models for regional mobility in Sub-Saharan Africa**

Amy Wesolowski^1,2^, Wendy Prudhomme O’Meara^3^, Nathan Eagle^1,4^, Andrew J. Tatem^5,6^, Caroline O. Buckee^1,2*^

^1^ Department of Epidemiology, Harvard School of Public Health, Boston, USA.

^2^ Center for Communicable Disease Dynamics, Harvard School of Public Health, Boston, USA.

^3^ Department of Medicine, Duke University and Duke Global Health Institute, Durham, USA.

^4^ Department of Computer Science, Northeastern University, Boston, USA.

^5^ Department of Geography and Environment, University of Southampton, Southampton, UK.

^6^ Fogarty International Center, National Institutes of Health, Bethesda, USA.

*Corresponding author: [cbuckee@hsph.harvard.edu](mailto:cbuckee@hsph.harvard.edu)

**Abstract**

Simple spatial interaction models of human mobility based on physical laws have been used extensively in the social, biological, and physical sciences, and in the study of the human dynamics underlying the spread of disease. Recent analyses of commuting patterns and travel behavior in high-income countries have led to the suggestion that these models are highly generalizable, and as a result gravity and radiation models have become standard tools for describing population mobility dynamics for infectious disease epidemiology. Communities in Sub-Saharan Africa may not conform to these models, however: physical accessibility, availability of transport, and cost of travel between locations may be variable and severely constrained compared to high-income settings, informal labor movements rather than regular commuting patterns are often the norm, and the rise of mega-cities across the continent has important implications for travel between rural and urban areas. Here, we first review how infectious disease frameworks incorporate human mobility on different spatial scales, and use anonymous mobile phone data from nearly 15 million individuals to analyze the spatiotemporal dynamics of the Kenyan population. We find that gravity and radiation models fail in systematic ways to capture human mobility measured by mobile phones: both severely overestimate the spatial spread of travel and perform poorly in rural areas, but each exhibits different characteristic patterns of failure with respect to routes and volumes of travel. Thus, infectious disease frameworks that rely on spatial interaction models are likely to misrepresent population dynamics important for the spread of disease in many African populations.

**Author Summary**

Human mobility underlies many social, biological, and physical phenomena including the spread of infectious diseases. Analyses in high-income countries have led to the notion that populations obey universal rules of mobility that are effectively captured by spatial interaction models. However, communities in Africa may not conform to these rules, since the availability of transport and geographic barriers may impose different constraints compared to high-income settings. We use anonymous mobile phone data from ~15 million subscribers to quantify different spatial and temporal scales of mobility within Kenya and test their performance with respect to this measurement of human travel. We find that standard models systematically fail to describe regional mobility in Kenya, with poor performance in rural areas. Epidemiological models that rely on these frameworks may therefore fail to capture important aspects of population dynamics driving disease spread in many African populations.

**Introduction**

Human mobility patterns underlie the spread of infectious diseases across spatial scales. Theoretical models of human mobility have been used to understand the spatial spread of influenza, cholera, and malaria, for example [[1-20](#_ENREF_1)] as well as to design targeted interventions [[1](#_ENREF_1),[5](#_ENREF_5),[20-22](#_ENREF_20)]. These models rely almost exclusively on two frameworks, the gravity model and the more recent radiation model, both of which were developed to describe regular commuting patterns in high-income settings [[23-26](#_ENREF_23)]. In the absence of easily available data on travel behavior, these models are increasingly also being applied to models of infectious disease dynamics in low and middle-income settings. Despite the need for robust epidemiological models in places like Sub-Saharan Africa, it remains unclear if gravity and radiation models adequately describe mobility in these populations.

Geographic constraints and economic drivers of travel may be substantially different in Sub-Saharan Africa than in high-income countries. Many African countries are experiencing rapid demographic changes and may have poor transportation infrastructure. Many populations remain subsistence farmers living in rural areas with limited economic opportunities, public resources, and infrastructure [[27](#_ENREF_27),[28](#_ENREF_28)]. Kenya exhibits many of these attributes, for example, including highly variable population density and substantial geographic diversity, ranging from the major urban commercial center of Nairobi (population density ~4,510/km^2^) to the pastoral communities in the northern part of the country (see Figure 1A). Only 7% of Kenyan roads are paved, often those in and out of the capital, as is common in many African countries. Despite these constraints, mobility in many parts of the continent has increased dramatically over the last decade [[29](#_ENREF_29)], with rural-to-urban migration, seasonal travel, and extensive travel for agricultural and casual laboring jobs forming important components of the emerging ecology of African populations [[30](#_ENREF_30)].

Data sources describing these travel patterns are rare, however [[31](#_ENREF_31),[32](#_ENREF_32)], so gravity (parameterized) and radiation (parameter-free) models offer intuitive and tractable analytical frameworks for describing human mobility patterns (Figure 1B, C). In their simplest forms both models rely on spatial population data as a proxy for the economic attractiveness of a place and assume a decay in the amount of travel with distance [[23](#_ENREF_23),[26](#_ENREF_26),[33](#_ENREF_33)]. In the standard gravity model, Euclidean distance is often used to inform this decay rate, whereas in the radiation model, an individual is likely to travel to the nearest location that offers an improvement in current working conditions (measured via population size), with decay described as a function of the populations and distance between locations. Extensions have been proposed to improve the standard gravity model to include more relevant driving factors of travel such as the percentage of the population that is male, economic activity measures, and land cover [[33](#_ENREF_33)]. Other formulations of the gravity model constrain the origin and destination travel and has been shown to outperform the standard gravity model [[25](#_ENREF_25)]. By definition, neither encompasses different types of journeys or different trip durations, which are often important aspects of travel for the spread of infectious disease.

Validating these frameworks, in low and middle-income settings in particular, remains challenging. Mobile phone data sets that are routinely collected by mobile operators provide an important new source of information about the dynamics of populations on an unprecedented scale, and provide an opportunity to measure human mobility directly for entire populations [[23](#_ENREF_23),[25](#_ENREF_25),[34-38](#_ENREF_34)]. The adoption of mobile phone technologies in Africa in particular has been rapid, providing the opportunity to study population dynamics of countries for the first time [[31](#_ENREF_31),[35](#_ENREF_35)]. Given the difficulties of obtaining and sharing mobile call data records (CDRs), however, it will be important to assess whether measured travel patterns in different regions support the use of gravity and radiation models in places without mobility data.

Here, we first review previous infectious disease models that have explicitly included a model of human mobility, and highlight the disparity between models and types of mobility quantified that are used for simulation versus those including epidemiological data. Next, we analyze CDRs from nearly 15 million subscribers in Kenya over the course of a year to test gravity and radiation models in this East African context. We test both gravity and radiation models in the context of Kenya, and show that both models fail to capture important aspects of mobility measured using CDRs, but in different ways. We then test their utility to describe travel over various trip durations and show differences in travel patterns between shorter and longer journeys. Finally, we highlight situations when each model outperforms the other and discuss a method to choose between models using the amount of travel.

**Results**

We first reviewed infectious disease models that explicitly include human mobility (Figure 2). Here, we focused only on models that represent the first time a particular formulation was used, and not subsequent versions of the same framework (see S1 Text for the inclusion criteria and overview of papers included, Table S1). We also included only papers that explicitly modeled both the disease dynamics and mobility patterns and have excluded papers that have not modeled both components (for example see [[4](#_ENREF_4),[10-17](#_ENREF_10)]). We found nineteen studies, eleven of which were purely simulated epidemiological models [[10-20](#_ENREF_10)] and eight of which included fits to epidemiological data [[1-9](#_ENREF_1)]. Although these studies analyzed a range of infectious diseases, nearly all simulation studies analyzed the spread of influenza in high-income countries using commuting as the relevant type of mobility (8 out of 11). The majority of examples used a gravity model (10 papers) [[2-8](#_ENREF_2),[10](#_ENREF_10),[13](#_ENREF_13),[17](#_ENREF_17),[18](#_ENREF_18)] and nearly all of the examples using a radiation model were for simulated disease dynamics only (2 papers) [[11](#_ENREF_11),[12](#_ENREF_12)]. The examples that were fit to disease data were more varied although the majority were from low-income countries (5) [[1](#_ENREF_1),[2](#_ENREF_2),[4](#_ENREF_4),[5](#_ENREF_5),[39](#_ENREF_39)] and described regional movement patterns (see Figure 2) [[1-5](#_ENREF_1)]. Thus, simple gravity model frameworks are very commonly used to understand the regional spread of infectious disease in low-income settings, highlighting the importance of testing their validity and generalizability.

To test the performance of gravity and radiation models in an African setting, we analyzed regional travel across Kenya from de-identified call detail records (CDRs) at the cell tower level from 14,816,521 individual subscribers between June 2008 and June 2009, representing 92% of mobile market share (data previously described in [[36](#_ENREF_36)]). We have previously used these data to quantify general mobility patterns as well as travel between locations of interest, and compared to census and travel survey data [[23](#_ENREF_23),[34](#_ENREF_34),[36](#_ENREF_36)]. Here we focused on regional movement patterns since this is the most common spatial resolution of mobility models used in conjunction with epidemiological data in low-income settings, and regional travel represents a major source of uncertainty in disease models currently. We calculated all journeys between 69 Kenyan districts over the course of one year, ignoring travel within districts. On this spatial scale, movements between districts within the timespan of one day are almost nonexistent (see S1 Text), so we used the most commonly used tower each day to approximate each subscriber’s location on a daily basis. We fit both an unconstrained gravity model and a radiation model to data, representing the total number of journeys of the course of the year between districts over the course of the data set (one year, see Materials and Methods). We fit a number of constrained gravity models, although these did not perform as well as the standard gravity model (see S1 Text). Here, we assume that travel measured by CDRs reflects “true” travel behavior, although it is likely to suffer from different types of bias, like any data on human mobility.

The models varied widely in their ability to capture observed travel patterns in and out of rural versus urban districts, as illustrated by travel from Nairobi and Garissa (Figure 3). Nairobi is densely populated (total population of district 3.4 million, 10% of the country’s population) encompassing the capital and major population and economic center in the country. Located in the middle of the country, this district is well connected by paved roads to the second largest city (Mombasa 1.2 million) as well as to western Kenya, where nearly half of the population resides. In this setting, both models were able to identify the primary destination locations accurately, although the radiation model predicted travel to a wider range of locations than observed in the CDRs (Figure 3A, B, C). Garissa, on the other hand, is a sparsely-populated low-income district bordering Somalia, and likely to be more similar to other rural areas in Africa than to high-income countries. For travel originating from Garissa, the predicted volumes and routes of travel were very different from empirical estimates (Figure 3D, E, F). Most strikingly, the gravity model predicted travel to a much wider range of destinations than observed, and the radiation model failed to identify the primary travel destination. These errors would be likely to lead models to over-estimate the spread of disease in the first case, and under-estimate disease importation into the capital city in the second.

The models diverged systematically in their predictions with regard to travel volume (Figure 4A, B) with the gravity model consistently over-predicting travel and the radiation model under-predicting travel (mean ratio of data to predicted results was 0.83 and 35.03, respectively, see S1 Text, Figure S1). Although the gravity model using Euclidean distance gave a better overall fit to the data than the radiation model (gravity model adjusted R^2^: 0.786, radiation model adjusted R^2^: 0.014, see S1 Text, Figure S2), this was due to the radiation model’s consistent failure to capture large volumes of human travel between major population centers. We hypothesized that one reason for the poor performance of both models in rural areas may be the impact of physical accessibility and road infrastructure on travel. This is likely to be particularly important in Sub-Saharan Africa, and adjusted measures of distance based on estimated travel times, as well as road distance, have been developed for these regions [[40](#_ENREF_40)]. We re-fit the parameters of the gravity model using road distance and travel times and found that Euclidean distance between district centroids provided the most accurate overall predictions of travel volume across a range of scenarios including the full dataset, travel to and from the capital, and large urban centers (reduction in deviance: 63%-87%). Interestingly, in rural areas road distance noticeably outperformed all other distance measures, suggesting that travel time estimates may not accurately reflect human behavior in these regions (see Figure 4C, S1 Text, Tables S2-S4).

We compared the distribution of errors from both models to identify “rules of thumb” for using gravity and radiation models to estimate volumes of travel (see Materials and Methods). We assumed the empirical error from each model should be normally distributed and categorized the travel routes that fall more than 2 standard deviations away from the mean (10% of routes, see Figure 5A, KS-statistic = 0.2481, p<0.001). In general, both models failed to adequately capture travel from rural areas of intermediate population density over shorter distances, especially in the western part of Kenya in the Rift Valley and Western provinces (Figure 5B, see S1 Text for further analysis, Table S5). Importantly, these rural regions of intermediate population density are likely to represent sizeable fractions of African populations; in Kenya these provinces where mobility models are systematically failing account for nearly 40% of the population (14 million individuals).

Neither the gravity nor the radiation model was consistently a superior choice, exhibiting different spatial patterns of performance (see Figure 5B), however in general the radiation model outperformed the gravity model for low amounts of travel and vice a versa. We calculated a naïve gravity factor, i.e. a gravity model without any parameters fit (pop_i * pop_j /d(i,j)) and performed a logistic regression to determine which flows were better predicted using each model (see Figure 5C, S1 Text for regression results using just populations or distance as covariates, Table S6 – adjusted R^2^= 0.5703, p<0.001). We observed a strong positive correlation between the gravity factor, which is proportional to the total amount of travel, and the odds of using a gravity model (Figure 5C). These results imply that a gravity model is more likely to capture the spread of disease between major urban centers, but a radiation model may be more appropriate for modeling rural-to-urban migration. In both cases, model performance varied substantially in different locations.

An important consideration for spatial models of infectious disease dynamics is the length of journeys, since it will help determine both the number of onward infections generated by an imported case and the risk of exposure to infection of a traveling individual. Gravity and radiation models do not make explicit assumptions about trip durations, but since they were primarily developed to model commuting patterns they may not be appropriate for understanding journeys of varying length. We therefore analyzed the spatial dimensions of human travel for trips of varying duration (see Table 1, Figure 6A) [[19](#_ENREF_19)] and the ability of each model to describe these different trips. As expected, the total number of trips between districts decreased as journey duration increased (see Figures 6, S3-S4). For example, the number of trips lasting between one and two weeks was on average two orders of magnitude greater than the number of trips lasting at least four months (see S1 Text). The major routes of travel also varied with the trip duration, with longer journeys being associated with increasing distances and larger population sizes at the destination, with Nairobi in particular becoming an increasingly important longer-term destination (see Figures 6B, S5-S6). We refit a separate gravity model for each duration of travel (note that we do not refit the radiation model since it is parameter free) (see Materials and Methods, S1 Text). This analysis highlights the difference in the major routes of travel, where the destination population parameter increased as the trip duration increased and the importance of distance in the model decreased (see Table 1).

**Discussion**

Our analysis suggests that gravity and radiation models do not adequately capture movements measured by mobile phones in rural and intermediate population density areas in Kenya, areas that are characteristic of many settings in Sub-Saharan Africa. These findings bring into question the universal applicability of these frameworks, and have important implications for estimating the risk of infectious disease importation, for example. Given the ubiquity of gravity and radiation models in epidemiological frameworks, we focused on validating these fundamental frameworks as opposed to examining more recent modifications [[24](#_ENREF_24),[41](#_ENREF_41)]. One important caveat is that we have compared these theoretical models to travel measured via mobile phones, which may be affected by variable ownership and usage patterns, particularly in poor or rural areas [37,42]. Nevertheless, mobile phone data currently represent one of the most direct ways to measure regional population dynamics, especially in low-income settings where commuting and travel survey data may be patchy [[42](#_ENREF_42),[43](#_ENREF_43)]. Here we have focused on the regional and inter-settlement spatial scales that can be measured using CDRs, but an important next step – particularly for infectious disease prediction – is to find appropriate data to examine the performance of gravity and radiation models on extremely local spatial and short temporal scales.

Future work devoted to developing a generalizable model that can accurately capture travel in Sub-Saharan Africa, particularly in rural areas with intermediate population densities, will be an important priority for the development of appropriate frameworks for a description of African population dynamics. As more mobile phone data sets become available, the generalizability of our results can be confirmed in other countries assuming mobile phone data provides a reasonable sample of the underlying population [[35](#_ENREF_35)]. Spatial interaction models can provide researchers with the ability to model population dynamics in low-income and data sparse settings, such as Sub-Saharan Africa. However the universality of these models is questionable, especially when describing rural travel in geographically and economically heterogeneous settings. Applications reliant on the underlying population dynamics derived from either model, such as understanding the spread of an infectious disease or the role of travel on economic activity, are likely to miss important routes and types of travel commonly found in Sub-Saharan Africa.

**Materials and Methods**

*Data Sources*

We analyzed anonymized mobile phone call data records (CDR) aggregated to the routing mobile phone tower level. These data were provided by the incumbent mobile phone (92% market share at the time of data acquisition) provider in Kenya and included the timings of calls and SMS from 14,816,512 subscribers from June 2008 - June 2009 (with February 2009 missing from the data set). As in previous studies [[23](#_ENREF_23),[34-36](#_ENREF_34)], subscribers represented in the CDRs as unique hashed IDs to protect their privacy. Twelve billion mobile phone communications were analyzed, recording activity at a total of 11,920 routing towers. All subscriber data was aggregated to the district level to further preserve anonymity. In the interest of protecting privacy, limited access to the anonymized data was made available to a select set of researchers.

*Quantifying travel patterns*

Each entry in a CDR contains an anonymized caller ID, anonymized receiver ID, date, duration, and tower routing number for both the caller and receiver. From the CDRs, the geographic location of the caller and receiver could be approximated based on the unique longitude and latitude coordinates for each mobile phone tower. Using the CDRs, a location for each subscriber every time they either made/received a call (or SMS) could be obtained. For each day in the data set, subscribers were assigned a single tower location [[35](#_ENREF_35),[36](#_ENREF_36)]. If the subscriber made at least one call on that day, then the location of the majority routing tower was assigned [[35](#_ENREF_35),[36](#_ENREF_36)]. If there was no majority routing tower, then for the most likely set of towers, a single tower was randomly chosen. If the subscriber had not made a call on that day, then the location of their most recent routing tower was assigned. This provided a time series of tower location for each subscriber on each day. As done in previous studies, trips are calculated by observing when a subscriber’s tower location has changed from the previous day for the entire data set (12 months of data) [[35](#_ENREF_35),[36](#_ENREF_36)]. We aggregated towers to the district-level based on the tower’s location and only trips between towers in different districts were considered to quantify regional movement patterns. In comparison to a number of other studies analyzing spatial interaction models and infectious disease dynamics, we did not focus on commuting patterns since we are describing regional movement patterns, e.g. movement within a country as opposed to within a single city, and few subscribers change districts between daytime and nighttime.

*Duration of travel*

We investigated the ability of these models to describe travel over various durations. Using the CDR, we were able to quantify both the number of trips between districts as well as the duration of those journeys (in days based on the daily location of each subscriber). For each trip between districts, we counted the number of days the subscriber spent in the visited district. Using the mobile phone data, we compared all travel (every trip between all pairs of districts over the entire data set) to journeys lasting various durations where trips were stratified into six separate groups (see Table 1). The category, All travel includes every trip taken between districts, regardless of trip duration. We grouped all trips lasting at least four months into a single category due to the length of the data set (in total 12 months of CDR data).

*Spatial Interaction Models*

| The gravity model is the most common spatial interaction model where the amount of travel (N_ij_) between two locations (i,j) is dependent on their populations (pop_i_, pop_j_) and the physical distance separating them (d(i,j)) [[26](#_ENREF_26),[35](#_ENREF_35),[36](#_ENREF_36)]: $N_{ij}=\frac{pop_{i}^{\alpha}pop_{j}^{\beta}}{d(i,j)^{\gamma}}k$ where the parameters $\alpha,\beta,\gamma,k$ are fit based on a Poisson distribution [[35](#_ENREF_35),[44](#_ENREF_44)]. We choose the fitting method based on Flowerdew [[44](#_ENREF_44)] where the amount of travel estimated using regression assuming a Poisson family.  The gravity model has been extensively used to model mobility in conjunctions with models of the spatial spread of infectious diseases [[2-8](#_ENREF_2),[10](#_ENREF_10),[13](#_ENREF_13),[17](#_ENREF_17),[18](#_ENREF_18)]. There have been a number of proposed additions and modifications to the gravity model including adding covariates such as the percentage of the population that is male [[33](#_ENREF_33)] or putting constraints on the number of trips [[25](#_ENREF_25)] such as the singly or doubly constrained model. Here, we fit the simplified since the model without covariates is the most commonly used for disease modeling [[1](#_ENREF_1),[19-25](#_ENREF_19),[29](#_ENREF_29),[33](#_ENREF_33),[34](#_ENREF_34)]. We also fit the origin singly constrained model, production singly constrained model, and doubly constrained model (see S1 Text). However, these non-constrained simplified gravity models outperformed these three models (increase in sum of square errors: non-constrained – 37.9%, origin constraint – 39.5%, destination constraint – 39.5%, and doubly constrained – 39.5%). We also fit separate gravity models to each set of data describing various trip durations (see Table 1 and S1 Text). |
| --- |

Recently, the radiation model has been proposed as an improvement on the gravity model [[23](#_ENREF_23)]. It draws its original inspiration from a gravity model, but is a stochastic process that only requires information on the population distribution and is parameter free. In this model, the average amount of travel (N_ij_) between two locations (i,j) is dependent upon their populations and the total population in the circle of radius r_ij_ centered at i where r_ij_ = d(i,j) (the circle population is s_ij_) : $<N_{ij}>=N_{i}(pop_{i}pop_{j}/(pop_{i}+s_{ij})(pop_{i}+pop_{j}+s_{ij})$. $N_{i}=pop_{i}(\frac{T_{c}}{T})$ where T_c_/T is the proportion of the population who travels. If no data is available to fit the radiation model, then T_c_/T is fixed and not fit. Here we fit this percentage to the actual data (see S1 Text) and the optimum value is T_c_/T = 1. Recently, extensions to this model have been proposed to reflect human behavior in employment choice using various functional forms [[24](#_ENREF_24)], however we have focused on the most commonly used model formulation.

*Distance Measures*

We analyzed three separate distance measures (Euclidean, road, and travel time between both district polygon centroids and district population weighted centroids) [[40](#_ENREF_40)]. Euclidean distance was measured as the straight-line distance between centroids. Road distance was measured using the road network data from the Kenya National Bureau of Statistics. These data with land cover data ([www.africover.org](http://www.africover.org)) and topography data (<http://srtm.csi.cgiar.org/>) were used to construct a ‘friction surface’ that was used to estimate travel time distances, following previously outlined methods [[40](#_ENREF_40)]. The travel time is based on a measure of friction between one location and another that takes into account land cover types, transport network and gradient. In general, this measure is thought to be more representative of the ease of human travel access across a landscape since it takes into account impedances to travel. Similar to previous methods, water bodies, land cover, slope and the road network datasets were combined on a 1km spatial resolution grid to empirically derive travel speeds [[40](#_ENREF_40)]. These travel speeds were assigned to each land use type and modified based on the topography to create a ‘friction surface’. This surface was used to estimate travel times between locations using least cost methods [[40](#_ENREF_40)], with those locations defined by population weighted centroids  (defined using high resolution population maps provided by the WorldPop Project: [www.worldpop.org.uk](http://www.worldpop.org.uk/)), where these centroids were automatically adjusted to be located to the nearest road.  Correlations between the measures can be found in the S1 Text.

*Choosing which data is poorly described by either model*

We calculated the error of each model as the difference between the data and estimated value (error = log(data) – log(predicted)). We took the standard assumption that these errors were normally distributed with mean 0 and standard deviation of 1. For any value not in the confidence interval, we suggest that caution should be taken when utilizing these estimates (see S1 Text, Table S5) (about 10% of the pairs of locations were eliminated).

*Choosing between models*

Of routes between districts that were well described by either model, we calculated a gravity factor, gm = pop_i * pop_j / dist(i,j) which is a equivalent to the gravity model without fitting any parameters, as a proxy for the amount of travel between locations. Using this covariate, we then performed a logistic regression to determine when the radiation model or gravity model produced lower errors compared to the actual data for these routes.

$logit_{gm}(p)=b_{0,gm}+{b_{1,gm}*X}_{gm}$ where $p$ is the probability of choosing a gravity model over a radiation model, $X_{gm}$ is the gravity factor we calculated. As this value increases, i.e. the amount of travel increases, the probability of choosing a gravity model over a radiation model increases (see Figure 5C).

**References**

1. Ferrari MJ, Grais RF, Bharti N, Conlan AJ, Bjornstad ON, et al. (2008) The dynamics of measles in sub-Saharan Africa. Nature 451: 679-684.

2. Gatto M, Mari L, Bertuzzo E, Casagrandi R, Righetto L, et al. (2012) Generalized reproduction numbers and the prediction of patterns in waterborne disease. Proc Natl Acad Sci U S A 109: 19703-19708.

3. Gog JR, Ballesteros S, Viboud C, Simonsen L, Bjornstad ON, et al. (2014) Spatial Transmission of 2009 Pandemic Influenza in the US. PLoS Comput Biol 10: e1003635.

4. Mari L, Bertuzzo E, Righetto L, Casagrandi R, Gatto M, et al. (2012) Modelling cholera epidemics: the role of waterways, human mobility and sanitation. J R Soc Interface 9: 376-388.

5. Metcalf CJ, Cohen C, Lessler J, McAnerney JM, Ntshoe GM, et al. (2013) Implications of spatially heterogeneous vaccination coverage for the risk of congenital rubella syndrome in South Africa. J R Soc Interface 10: 20120756.

6. Tatem AJ, Smith DL (2010) International population movements and regional Plasmodium falciparum malaria elimination strategies. Proc Natl Acad Sci U S A 107: 12222-12227.

7. Xia Y, Bjornstad ON, Grenfell BT (2004) Measles metapopulation dynamics: a gravity model for epidemiological coupling and dynamics. Am Nat 164: 267-281.

8. Balcan D, Hu H, Goncalves B, Bajardi P, Poletto C, et al. (2009) Seasonal transmission potential and activity peaks of the new influenza A(H1N1): a Monte Carlo likelihood analysis based on human mobility. BMC Med 7: 45.

9. Wang L, Li X, Zhang YQ, Zhang Y, Zhang K (2011) Evolution of scaling emergence in large-scale spatial epidemic spreading. PLoS One 6: e21197.

10. Balcan D, Colizza V, Goncalves B, Hu H, Ramasco JJ, et al. (2009) Multiscale mobility networks and the spatial spreading of infectious diseases. Proc Natl Acad Sci U S A 106: 21484-21489.

11. Dalziel BD, Pourbohloul B, Ellner SP (2013) Human mobility patterns predict divergent epidemic dynamics among cities. Proc Biol Sci 280: 20130763.

12. Tizzoni M, Bajardi P, Decuyper A, Kon Kam King G, Schneider CM, et al. (2014) On the use of human mobility proxies for modeling epidemics. PLoS Comput Biol 10: e1003716.

13. Truscott J, Ferguson NM (2012) Evaluating the adequacy of gravity models as a description of human mobility for epidemic modelling. PLoS Comput Biol 8: e1002699.

14. Vazquez-Prokopec GM, Bisanzio D, Stoddard ST, Paz-Soldan V, Morrison AC, et al. (2013) Using GPS technology to quantify human mobility, dynamic contacts and infectious disease dynamics in a resource-poor urban environment. PLoS One 8: e58802.

15. Watts DJ, Muhamad R, Medina DC, Dodds PS (2005) Multiscale, resurgent epidemics in a hierarchical metapopulation model. Proc Natl Acad Sci U S A 102: 11157-11162.

16. Meloni S, Perra N, Arenas A, Gomez S, Moreno Y, et al. (2011) Modeling human mobility responses to the large-scale spreading of infectious diseases. Sci Rep 1: 62.

17. Merler S, Ajelli M (2010) The role of population heterogeneity and human mobility in the spread of pandemic influenza. Proc Biol Sci 277: 557-565.

18. Mills HL, Riley S (2014) The spatial resolution of epidemic peaks. PLoS Comput Biol 10: e1003561.

19. Poletto C, Tizzoni M, Colizza V (2012) Heterogeneous length of stay of hosts' movements and spatial epidemic spread. Sci Rep 2: 476.

20. Keeling MJ, Danon L, Vernon MC, House TA (2010) Individual identity and movement networks for disease metapopulations. Proc Natl Acad Sci U S A 107: 8866-8870.

21. Cummings DA, Irizarry RA, Huang NE, Endy TP, Nisalak A, et al. (2004) Travelling waves in the occurrence of dengue haemorrhagic fever in Thailand. Nature 427: 344-347.

22. Ferguson NM, Keeling MJ, Edmunds WJ, Gani R, Grenfell BT, et al. (2003) Planning for smallpox outbreaks. Nature 425: 681-685.

23. Simini F, Gonzalez MC, Maritan A, Barabasi AL (2012) A universal model for mobility and migration patterns. Nature 484: 96-100.

24. Simini F, Maritan A, Neda Z (2013) Human mobility in a continuum approach. PLoS One 8: e60069.

25. Yang Y, Herrera C, Eagle N, Gonzalez MC (2014) Limits of predictability in commuting flows in the absence of data for calibration. Sci Rep 4: 5662.

26. Zipf G (1946) The P1P2/D hypothesis: On the inter-city movement of persons. Am Sociol Rev 11: 677–686

27. Kessides C (2005) The urban transition in Sub-Saharan Africa: Implications for economic growth and poverty reduction. Africa Region Working Paper Series No 97 The World Bank

28. Foster V, Briceno-Garmendia, C (2010) Africa’s infrastructure: A time for transformation. . World Bank.

29. Bryceson D. F. MDAC, Mbara T. C., Kibombo R., Davis A. S. C., Howe J. D. G. F. (2003) Sustainable livelihoods, mobility, and access needs. TRL Report TRL544.

30. Prothero RM (1977) Disease and mobility: a neglected factor in epidemiology. Int J Epidemiol 6: 259-267.

31. Pindolia DK, Garcia AJ, Wesolowski A, Smith DL, Buckee CO, et al. (2012) Human movement data for malaria control and elimination strategic planning. Malar J 11: 205.

32. Tatem AJ (2014) Mapping population and pathogen movements. Int Health 6: 5-11.

33. Henry S, Boyle, P., Lambin, E. F. (2002) Modeling inter-provincial migration in Burkina Faso, West Africa: the role of socio-demographic and environmental factors. App Geo: 115-136

34. Gonzalez MC, Hidalgo CA, Barabasi AL (2008) Understanding individual human mobility patterns. Nature 453: 779-782.

35. Wesolowski A, Buckee CO, Pindolia DK, Eagle N, Smith DL, et al. (2013) The use of census migration data to approximate human movement patterns across temporal scales. PLoS One 8: e52971.

36. Wesolowski A, Eagle N, Tatem AJ, Smith DL, Noor AM, et al. (2012) Quantifying the impact of human mobility on malaria. Science 338: 267-270.

37. Wesolowski A, Stresman G, Eagle N, Stevenson J, Owaga C, et al. (2014) Quantifying travel behavior for infectious disease research: a comparison of data from surveys and mobile phones. Sci Rep 4: 5678.

38. de Montjoye YA, Smoreda, Z., Trinquart, R., Ziemlicki, C., Blondel, V.D. (2014) D4D-Senegal: The Second Mobile Phone Data for Development Challenge. <http://www.arXiv.org>.

39. Stoddard ST, Forshey BM, Morrison AC, Paz-Soldan VA, Vazquez-Prokopec GM, et al. (2013) House-to-house human movement drives dengue virus transmission. Proc Natl Acad Sci U S A 110: 994-999.

40. Linard C, Gilbert M, Snow RW, Noor AM, Tatem AJ (2012) Population distribution, settlement patterns and accessibility across Africa in 2010. PLoS One 7: e31743.

41. Schneider CM, Belik V, Couronne T, Smoreda Z, Gonzalez MC (2013) Unravelling daily human mobility motifs. J R Soc Interface 10: 20130246.

42. Wesolowski A, Eagle N, Noor AM, Snow RW, Buckee CO (2012) Heterogeneous mobile phone ownership and usage patterns in Kenya. PLoS One 7: e35319.

43. Wesolowski A, Eagle N, Noor AM, Snow RW, Buckee CO (2013) The impact of biases in mobile phone ownership on estimates of human mobility. J R Soc Interface 10: 20120986.

44. Flowerdew R, Aitkin M (1982) A method of fitting the gravity model based on the Poisson distribution. J Reg Sci 22: 191-202.

**Figure Legends**

**Fig. 1 The country of study and gravity and radiation model example results.** A) A population map of Kenya with district boundaries (grey) and major roads (black). For two districts, a schematic representation of B) a gravity model and C) a radiation model. The gravity model is based on the populations of the destination and origin as well as the distance between these locations. The radiation model is based on the destination and origin’s populations as well as the total population within a circle centered at the origin. This model is based on the premise that individuals living in a certain home location will consider the number of job opportunities (measured as the destination’s population proportional to the resident population) and will travel to the closest destination that would offer better benefits than the resident location.

**Fig. 2 A summary of previously published papers incorporating human mobility models and infectious disease dynamics.** We reviewed nineteen papers that either simulated (simulated) disease dynamics or used epidemiological data (data). These papers covered a range of infectious diseases (see Table S1). For each paper we identified the type of mobility model, disease model, location (high, low, or both high and low income country), and the type of movement quantified. Mobility models were classified as a gravity model, radiation model, a spatial transmission kernel, a network, or a risk surface. Disease models included a metapopulation model, metapopulation type model with stochastic fadeouts, time spent at locations with different risks of becoming infected, an individual based model (IBM), and a network. For each paper, the location of either the mobility and/or disease data determined the location of the paper with countries separated as high income or low income. Papers that focused on global disease spread were classified as both high and low income. Mobility was classified as either commuting, regional, or global movement patterns. If the paper did not explicitly state the type of mobility included in the paper the type of mobility was discerned from the spatial resolution of the data. Regional movement includes mobility between political admin units that are larger than a city, in general. If a paper included both global movements, such as airline flights, and localized commuting, then the paper was classified as global. We included papers describing various infectious diseases including cholera, malaria, dengue, measles, pertussis, rubella, influenza, and foot and mouth disease.

**Fig. 3 The actual and predicted amounts of travel from two districts.** The A,D) actual amount of travel, predicted amounts of travel from the B,E) gravity model and C,F) radiation model are shown for two districts. For each map, a continuous density surface was constructed showing the relative spatial distribution of travel. The bar plot shows the amount of travel to all other districts from the district with values displayed in increasing order of distance. Nairobi A,B,C) is the most populated district in the country and includes the capital. In all three figures, the majority of travel (shown in dark blue) is to neighboring locations. The radiation model estimates more travel to the rest of the country than the data or gravity model. Garissa D,E,F) is a rural district bordering Somalia. The majority of actual travel occurs to Nairobi, which the radiation model did not capture. The gravity model was able to predict a large amount of travel to Nairobi, but greatly over predicts travel to the rest of the country.

**Fig. 4 The results of fitting each spatial interaction model.** A) The predicted results from both gravity and radiation models. The gravity model (shown in blue) predicts larger amounts of the total volume of travel over the course of the data set (ratio of predicted values to data – mean: 12, 95% quantile interval: 0.34-43) than the data whereas the radiation model (shown in red) underpredicts the volume of travel (ratio of predicted values to data – mean: 0.5, 95% quantile interval: 0.0066-1.7). B) The ratio of predicted versus actual data from both models versus distance. For both models, the predictions over short distances were worse than over longer distances. C) We re-fit gravity models using Euclidean distance (red), travel times (blue) and road distance (green) between district centroids (circle) and population-weighted district (square) centroids. The reduction in deviance of these models is shown. In general, Euclidean distance based gravity models outperformed all other distance measures, except for travel between rural areas. For this type of travel, road distance outperformed Euclidean distance (Euclidean distance – reduction in deviance: 63%, road distance – reduction in deviance: 72%, see S1 Text).

**Fig. 5 The utility of each model to describe travel in various settings.** A) For travel between all pairs of locations, we compared the error in the actual versus predicted amount of travel. If this error was not within defined bounds (outside of the dotted black lines) (see Materials and Methods), we determined that both models do not adequately describe this travel (shown in black). For the remaining volumes and routes of travel, we determined if the gravity model (blue) or the radiation model (red) performed better to identify which model should be used in various settings. For example, the radiation model does much better than the gravity model predicting low volumes of travel and vice versa (shown in red versus blue). B) A schematic highlighting the situations when a radiation model is preferred over a gravity model and when caution should be taken using the predicted results of either model. Scenarios to use a gravity model over a radiation model include: travel to and from a major population centre, over short distances, and when predicting large volumes of travel. A radiation model should be used over a gravity model when describing travel between rural areas and low volumes of travel. Caution should be taken using either model if the travel is between locations of intermediate rural population and over short distances. C) We performed a logistic regression to determine when to use each model for various amounts of travel (a gravity factor). As the amount of travel increases, the number of times the gravity model outperforms the radiation model increases. For examples for the lowest amounts of travel 43% of the time a radiation model if preferable to a gravity model. In contrast for the largest amount of travel, all of these routes (100%) were better predicted with a gravity model than a radiation model.

**Fig. 6 The impact of the duration of journeys on travel between districts.** A) For travel between pairs of locations, we compared the number of trips (log) versus the distance (km) for all journeys (grey) and the best fit lines for trips lasting up to between one and two weeks (red), between two weeks and one month (blue), between one and two months (green), between two and three months (orange), between three and four months (purple), and trips lasting four months or more (yellow). As the duration of journeys increased, the amount of travel between districts decreased. B) For all trips including any trip duration (red) and those lasting 4+ months (grey), the top 5% of of routes are shown based on the total amount of travel. For trips lasting short durations, there is a large amount of travel between nearby districts. For trips lasting long durations, the majority of these top routes are to/from major cities including Nairobi and Mombasa.

**Table 1 The gravity model parameters and fit for trips lasting various durations.** For each duration of travel we measured, the gravity model parameters (with CI in parentheses) and fit (the percentage reduction in deviance) is shown. As the duration of travel increases, the destination population (Dest Pop) parameter increases and the distance (Dist) parameter decreases.

| Variable | Description | Origin Pop ($\alpha$) | Dest Pop ($\beta$) | Dist ($\gamma$) | Constant (k) | Reduc  Dev (%) |
| --- | --- | --- | --- | --- | --- | --- |
| All | Every trip regardless of duration | 1.22  (1.22, 1.22) | 1.22  (1.22, 1.22) | 2.05  (2.05, 2.05) | -20.06  (-20.6, -20.61) | 80 |
| Week | One – two weeks | 0.95  (0.95, 0.95) | 0.905  (0.905, 0.905) | 1.73  (1.73, 1.73) | -12.9  (-12.9, -12.9) | 60 |
| Biweek | Two weeks – one month | 0.903  (0.902, 0.904) | 0.907  (0.906, 0.907) | 1.58  (1.58, 1.58) | -14.8  (-14.9, -14.8) | 54 |
| Month | One – two months | 0.907  (0.906, 0.908) | 1.086  (1.085, 1.087) | 1.47  (1.47, 1.48) | -17.6  (-17.7, -17.6) | 54 |
| Month 2 | Two –three months | 0.91  (0.91, 0.91) | 1.39  (1.39, 1.4) | 1.33  (1.33, 1.33) | -22.4  (-22.4, -22.4) | 55 |
| Month 3 | Three –four months | 0.902  (0.9, 0.904) | 1.81  (1.8, 1.81) | 1.19  (1.19, 1.19) | -29.02  (-29.0, -29.1) | 57 |
| Month 4+ | Four+ months | 0.9  (0.89, 0.902) | 1.94  (1.93, 1.94) | 1.11  (1.11, 1.12) | -31.64  (-31.6, -31.7) | 57 |

**Supplementary Information**

**S1 Text: The supplementary information text.**

**Table S1: A summary of the papers analyzed by disease.** Papers that included epidemiological disease data are labeled ‘D’ whereas those that completely simulated disease dynamics are labeled ‘S’.

**Table S2: The gravity model parameters fit for subsets of the data.**

**Table S3A: The reduction in deviance from a gravity model for subsets of the data using various distance measures.** For the full data set, data including travel to/from: Nairobi, cities, and not including travel to/from: Nairobi, cities, the reduction in deviance (%) from fitting a gravity model is shown. For each distance measure, both population weighted centroids and non-population weighted centroids were calculated.

**Table S3B: The reduction in deviance from a gravity model for subsets of the data using various distance measures.** For data including travel between or to/from very and moderately rural areas, the reduction in deviance (%) from fitting a gravity model is shown. For each distance measure, both population weighted centroids and non-population weighted centroids were calculated.

**Table S4A: The Sorsensen-Dice coefficient for subsets of the data using various distance measures.** For data including travel between or to/from very and moderately rural areas, the Sorsensen-Dice coefficient from fitting a gravity model is shown. For each distance measure, both population weighted centroids and non-population weighted centroids were calculated.

**Table S4B: The Sorsensen-Dice coefficient for subsets of the data using various distance measures.** For data including travel between or to/from very and moderately rural areas, the Sorsensen-Dice coefficient from fitting a gravity model is shown. For each distance measure, both population weighted centroids and non-population weighted centroids were calculated.

**Table S5: The ability of each model The ability of each model to capture various situations % (N).**

**Table S6: The regression results predicting when to use a gravity model or radiation model.** We fit a number of logistic regression equations using distance, the origin population, or destination population as the explanatory variable. For each regression equation (see above equations), the coefficients, intercept, and model fit is shown (percentage reduction in deviance and adjusted R^2^ value).

**Figure S1: The relationship between empirical and predicted data to various factors.** The relationship between the ratio of the predicted versus actual data is shown compared to the population A) of the origin, B) of the destination, and C) the distance between the origin and destination. The gravity model consistently overpredicted travel, whereas the radiation model consistently underpredicted travel. For both the origin and destination population, there was no clear bias in the ability of each model to predict the volume of travel, although both models predicted more accurate estimates as the distance increased. D) The relationship between distance the amount of travel (log) with the trend line from predictions from the data (black), the gravity model (blue), and radiation model (red).

**Figure S2: The fit of each spatial interaction model.** Using Euclidean distance, we compared the estimated versus empirical amount of travel between areas of varying population size and distance from the radiation and gravity models. We calculated a Sorensen-Dice coefficient to measure the difference between predicted and total volumes of travel. The coefficient values from a A) gravity model and B) radiation model are shown highlighting the better model performance of the gravity model. Both models performed well at predicting travel to nearby highly populated districts. In general, C) the gravity model outperformed the radiation model. We next compared the ability of both models to predict the relative amount of travel D) gravity model, F) radiation model. Both models performed better at predicting relative travel than the total volume of travel with G) the radiation model often outperforming the gravity model.

**Figure S3: The number of trips versus the distance between the origin and destination districts for various trip durations.** For all durations of travel, we compared the number of trips versus the Euclidean distance between the origin and destination. For all trip durations, the frequency of trips decays with geographic distance. As the duration of travel increases, the frequency of journeys decreases.

**Figure S4: The relationship between the ratio of the predicted versus actual data is shown compared to the distance between the origin and destination.** For each group of travel based on trip duration, we compared the gravity model predicted values (using Euclidean distance) versus the Euclidean distance between districts (in kilometers). In general, each gravity model over predicted travel and produced the most accurate estimates (log(predict/data) near 0) for travel over short geographic distances.

**Figure S5: The top 5 percent of routes for various trip durations.** For each duration of travel, the top five percent of routes are shown. For trips lasting shorter durations, the most traveled routes are often to nearby districts. However, as the trip duration increases the most travel routes often include a major city such as Nairobi or Mombasa.

**Figure S6: The top 200 routes of travel for various trip durations.** Similar to Figure S5, we have plotted the top 200 routes of travel for various trip durations. As the trip duration increased, the most traveled routes often include a major city such as Nairobi or Mombasa.
